# A Short Peptide Hydrogel with High Stiffness Induced by 3_10_‐Helices to β‐Sheet Transition in Water

**DOI:** 10.1002/advs.201901173

**Published:** 2019-09-10

**Authors:** Shu Hui Hiew, Harini Mohanram, Lulu Ning, Jingjing Guo, Antoni Sánchez‐Ferrer, Xiangyan Shi, Konstantin Pervushin, Yuguang Mu, Raffaele Mezzenga, Ali Miserez

**Affiliations:** ^1^ Center for Biomimetic Sensor Science School of Materials Science and Engineering Nanyang Technological University Singapore 639798 Singapore; ^2^ School of Biological Sciences Nanyang Technological University Singapore 637551 Singapore; ^3^ Department of Health Sciences & Technology ETH Zurich Zurich CH‐8092 Switzerland

**Keywords:** β‐sheet transition, molecular dynamics (MD) simulations, NMR spectroscopy, peptide hydrogels, suckerin

## Abstract

Biological gels generally require polymeric chains that produce long‐lived physical entanglements. Low molecular weight colloids offer an alternative to macromolecular gels, but often require ad‐hoc synthetic procedures. Here, a short biomimetic peptide composed of eight amino acid residues derived from squid sucker ring teeth proteins is demonstrated to form hydrogel in water without any cross‐linking agent or chemical modification and exhibits a stiffness on par with the stiffest peptide hydrogels. Combining solution and solid‐state NMR, circular dichroism, infrared spectroscopy, and X‐ray scattering, the peptide is shown to form a supramolecular, semiflexible gel assembled from unusual right‐handed 3_10_‐helices stabilized in solution by π–π stacking. During gelation, the 3_10_‐helices undergo conformational transition into antiparallel β‐sheets with formation of new interpeptide hydrophobic interactions, and molecular dynamic simulations corroborate stabilization by cross β‐sheet oligomerization. The current study broadens the range of secondary structures available to create supramolecular hydrogels, and introduces 3_10_‐helices as transient building blocks for gelation via a 3_10_‐to‐β‐sheet conformational transition.

## Graphical abstract

**A short biomimetic peptide** self‐assembles into a hydrogel by a conformational transition from 3_10_‐helices to antiparallel β‐sheets. The eight‐residue peptide—derived from squid sucker ring teeth proteins—assembles into hydrogels with concentration‐dependent and tunable modulus without cross‐linking agents or chemical modifications. This study discusses the gelation dynamics and introduces 3_10_‐helices as transient building blocks for peptide hydrogels.

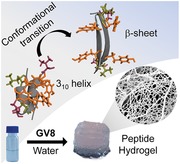

## 1 Introduction

Peptide hydrogels are increasingly explored for biomedical applications such as wound healing patches,^1^, ^2^ cell culture scaffolds for tissue engineering,^3^ drug delivery vehicles,^4^ or as substrates to study stem cell differentiation.^5^ Peptides are particularly attractive as building blocks for hydrogels because: (i) their chemical structure and polydispersity is fully controlled, (ii) they exhibit high biocompatibility,^6^ and (iii) their degradation products (amino acids) are readily cleared or reabsorbed by the metabolism.^6^ In addition, bioactive functionality can be achieved: for example RGD peptides can be incorporated into the peptide sequence^7^ to promote cell recognition or the peptide can be chemically modified with fluorescent probes and dye reporters^8^ or with functional groups to promote subsequent crosslinking reactions.^9^

In recent years, the ability to tune the gels' mechanical properties has become an increasingly important factor in the consideration of gel design.^10^ While some hydrogels employ cross‐linking to vary the elastic properties,^11^ others can be altered by varying the amount of salt in the gelation buffer or by adjusting the peptide concentration.^12^ In many cases, short peptide‐based hydrogels are assembled from β‐sheets, β‐hairpins, or coiled‐coil α‐helices.^13^, ^14^ Some peptides employ organic solvents to trigger gelation or toxic chemicals for cross‐linking, which is not ideal from a biocompatibility perspective.^11^, ^15^ Hence peptide hydrogels assembled from uncommon secondary structural constructs may expand the existing peptide hydrogel libraries and have the potential to provide new characteristics, such as a broader range of moduli and water‐based gelation.

Here, we present a short 8‐residue peptide (Ac‐**GLYGGYGV**‐NH_2_ hereafter called **GV8**) that gels in water. **GV8** exhibits a tunable, concentration‐dependent mechanical response with ≈25‐fold variation in storage modulus (*G′*) and a maximum value reaching 35.5 kPa that places it among the stiffest protein‐based hydrogels. The peptide sequence originates from suckerin proteins discovered in the sucker ring teeth (SRT) of the jumbo squid.^16^, ^17^, ^18^ Suckerins are a protein family with a characteristic modular primary structure consisting of long Gly‐rich modules previously assumed to form mostly unordered domains, which are intervened by smaller Ala‐ and His‐rich modules that self‐assemble into stiffer β‐sheet elements.^17^, ^18^ However, a recent NMR study indicated that the Gly‐rich domain can also form β‐sheets stabilized by aromatic side‐chain interactions.^19^ **GX8** peptides (where X = Val, Leu, and Phe) are specifically located in the Gly‐rich modules of suckerin‐19 with a high occurrence of 13 copies. Combining circular dichroism (CD), Fourier Transform Infrared Spectroscopy (FTIR), solution and solid‐state NMR, wide‐angle X‐ray scattering (WAXS), and molecular dynamics (MD) simulations, we find that **GV8** peptide forms 3_10_ helices in solution and undergoes a conformation change into antiparallel β‐sheets during gelation.

## 2 Results and Discussion

### 2.1 Peptide Gelation

We obtained the **GV8** peptide hydrogel by simple incubation of the peptide in deionized (DI) water, with gelation occurring at peptide concentrations ranging from 10 to 20 × 10^−3^ m and a concentration‐dependent gelation time between 5 and 9 h. The minimal critical gelation concentration (*C*_gc_) in water was 10 × 10^−3^ m, below which we did not observe gelation. We monitored the gelation kinetics by measuring the absorbance (OD_550nm_) of the peptide solutions at 550 nm (Figure S1, Supporting Information), whereby OD_550nm_ increased during the gelation process and plateaued once gelation was complete.^20^ We also attempted to mutate the C‐terminus Val residue with Leu (**GL8**), Ala (**GA8**), Phe (**GF8**), Ser (**GS8**), Lys (**GK8**), or Ile (**GI8**), but these peptides were not able to form gels in water, illustrating the key role of terminal Val in gelation as corroborated by NMR studies. **GF8** and **GI8** peptides remained in solution with some aggregates observed over time, whereas **GL8** self‐assembled into large (mm‐size) and stiff beads (Figure S2, Supporting Information).

### 2.2 Macro‐ and Microgel Structure

**Figure 1:**
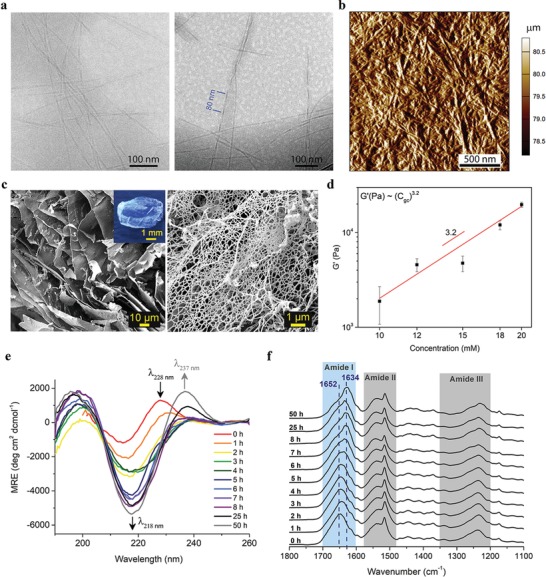
Structural features and physico‐chemical properties of **GV8** peptide hydrogel observed with time‐series spectroscopy measurements during gelation. a) Cryo‐EM images of **GV8** hydrogel fibrils and their twisted morphology (right image) with average periodicity of ≈80 nm. b) AFM amplitude profile of dried **GV8** hydrogel with fibers of ≈6–10 nm height. c) SEM images of **GV8** hydrogel cross‐section revealing sheet‐like morphology (left image) made of fibers (right image). A photograph of the hydrogel is shown in the inset. d) Scaling law plot of plateau *G*′ versus peptide concentration. e) CD and f) FTIR spectra recorded over 50 h indicate significant increase in β‐sheet content (intensity increase at λ_218 nm_ and *ν∼*_1634 cm_^−1^, respectively).

We then examined the morphology and topology of **GV8** hydrogel by Cryo‐Electron Microscopy (Cryo‐EM), atomic force microscopy (AFM), and scanning electron microscopy (SEM). 20 × 10^−3^ m **GV8** peptide solution was incubated for 3 h prior to blotting and vitrification to preserve the natural nanostructure of the sample in hydrated conditions^21^ for Cryo‐EM imaging. Long fibers less than 10 nm wide were observed (**Figure** **1**a) with consistent twisted morphologies and average periods of ≈80 nm along the fibers. AFM imaging was performed on a thin layer of dried gel, revealing a surface topology of a network of fibers (Figure 1b), and the height profile (Figure S3, Supporting Information) revealed fibers of ≈5–10 nm in height thereby matching the Cryo‐EM observations. Since drying and conventional lyophilization causes the hydrogel structure to collapse, samples for SEM were prepared by snap‐freezing **GV8** hydrogel in liquid N_2_ for at least 5 min followed by cryo‐fracture and immediate lyophilization to obtain representative cross‐sections. SEM imaging revealed a porous structure (Figure 1c, left) constructed by sheet‐like structures and closer examination indicated that the sheets were formed by a fibrous network of peptides (Figure 1c, right).

### 2.3 Rheology Characterization

**Figure 2:**
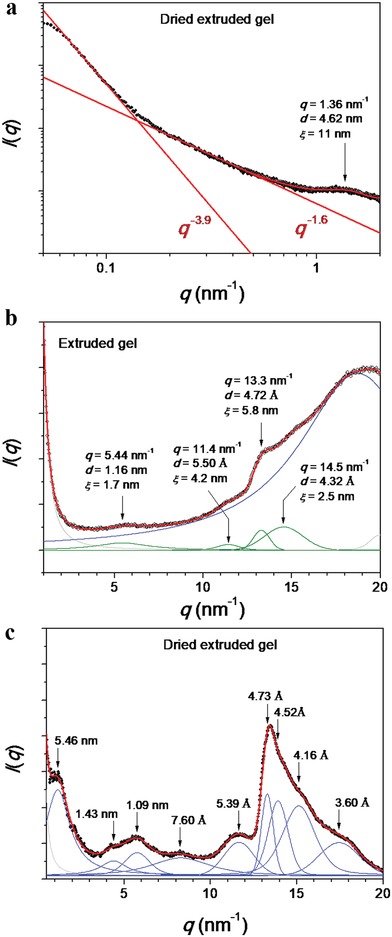
SAXS and WAXS patterns for the **GV8** peptide extruded gel together with the fitting curve (red) and peaks assignment. a) SAXS profile for dried extruded **GV8** gel. WAXS patterns of **GV8** gel in the b) hydrated state and in the c) dried state.

The hydrogel exhibited robust mechanical properties and could readily be manipulated and sectioned into thin slices (Movie S1, Supporting Information). In order to characterize the gel's mechanical properties, we prepared **GV8** hydrogels with peptide concentrations *C*_gc_ ranging from 10 to 20 × 10^−3^ m and conducted oscillation frequency sweeps at 0.25% shear strain (Figure S4, Supporting Information). The shear storage modulus (*G′*) exhibited a scaling power law as a function of peptide concentration (*G′* vs *C*_gc_) with a power law index of 3.2 (Figure 1d), allowing us to tune the storage modulus ≈25‐fold over a narrow range of peptide concentration. We note that this power law index is significantly higher than the “universal” scaling law determined for protein‐based semiflexible networks^22^, ^23^, ^24^ where *G′* scales as *C* ^11/5^ or for cross‐linked gels that exhibit a *G′* ∝ *C* ^2.5^ scaling law.^25^ Instead, this behavior is well captured by the fractal gel model, $G^{\prime }\propto C_{\mathrm{gc}}^{\left(\left(3+d_{b}\right)/\left(3-d_{f}\right)\right)}$, where *d*_b_ is the fractal dimension of the connecting chain and *d*_f_ is the dimensionality of the repeating fractal cluster. Taking *d*_f_ = 1.6 for the fractal dimension as obtained by small angle X‐ray scattering (SAXS, **Figure** **2**a) yields *d*_b_ = 1.5, which is a typical value of heat set protein gels.^26^ This behavior suggests that gelation does not proceed by entanglement of long fibrils, but rather by growth of shorter fibrillar clusters and conformational transition as evidenced by NMR and WAXS measurements described later. In line with this picture, **GV8** did not strain‐stiffen, likely because the chain length is shorter than in conventional biological gels. In quantitative terms, the maximum shear modulus of **GV8** of 35.5 kPa is on par with the stiffest, non‐crosslinked short peptide‐based hydrogel containing only natural amino acid residues.^12^ A wide range of moduli have been reported for short peptide hydrogels^10^, ^27^, ^28^, ^29^ with stiffest gels reached in gels containing modified amino acids, synthetic functional groups, or which have been crosslinked.^30^, ^31^ The ability to tune the stiffness from 1.3 to 35.5 kPa is particularly appealing for stem cell differentiation studies since gel stiffness has been well‐documented to govern cell adhesion and regulation based on the substrate's mechanical feedback,^5^, ^32^, ^33^, ^34^ with stiffness values in the range 0.1–1, 8–17, and 25–40 kPa sought after for neurogenic, myogenic, and osteogenic differentiation, respectively. The advantage of our **GV8** hydrogel for such applications is that its stiffness can be modulated solely by varying the peptide concentration without any additional chemical modifications.

### 2.4 Circular Dichroism and FTIR Spectroscopy

Next, we conducted time‐dependent CD and attenuated total reflection Fourier transform infrared spectroscopy (ATR‐FTIR) measurements on 20 × 10^−3^ m **GV8** from its initial solution state until its postgelation state in order to reveal secondary structural changes during self‐assembly (Figure 1e,f). At time 0 h, the CD spectrum consisted of a minimum at 215 nm and two maxima at 200 and 228 nm (Figure 1e). The bands at 215 and 228 nm are attributed to the resultant exciton couplet of Tyr–Tyr exciton interaction of their π→π* transitions, also known as CD Cotton effect^35^, ^36^ indicating interaction between the Tyr aromatic chromophores,^37^, ^38^ as also corroborated by our 2D NMR data. Over the course of gelation (1–5 h), the minimum shifted to 218 nm with a significant increase in intensity, and the maximum at 228 nm diminished, thereby transitioning to β‐sheet secondary structure. The CD spectra remained constant after 5 h in agreement with our OD_550nm_ measurements of 20 × 10^−3^ m **GV8** peptide (Figure S1, Supporting Information), whereby the absorbance plateau onset at 5 h indicated that gelation was complete without any significant structural changes after 5 h. Upon further incubation postgelation (25 and 50 h), a new maximum appeared at 237 nm and can be assigned to aromatic transitions of the Tyr residues,^39^ which we postulate is related to the conformational transition of **GV8** peptide during gelation.

ATR‐FTIR was performed on dried 20 × 10^−3^ m **GV8** hydrogels incubated over the same time points as in CD studies. The samples were snap‐freezed in liquid N_2_ to arrest the structural assembly of the peptides at stipulated time points. Amide I bands were deconvoluted and peaks assigned to β‐sheets, unordered regions, helices and turns, or 3_10_‐helices (Figure S5, Supporting Information).^40^, ^41^ β‐turns and 3_10_‐helices were grouped together in our assignments as they are structurally alike^42^, ^43^ with similar hydrogen bond strengths, and hence close frequency positions within the Amide I band. Over the course of gelation, the Amide I band maximum at *ν˜*_max_ = 1652 cm^−1^ shifted to 1634 cm^−1^ (Figure 1f), confirming secondary structural change towards β‐structures. Semiquantitative analysis by deconvolution of Amide I band (Table S1, Supporting Information) indicated that the initial dominating secondary structures of **GV8** peptide were turns and/or 3_10_, whereas in the gel state β‐sheet structures were the most abundant (≈65 % at 50 h).

### 2.5 Solution NMR

**Figure 3:**
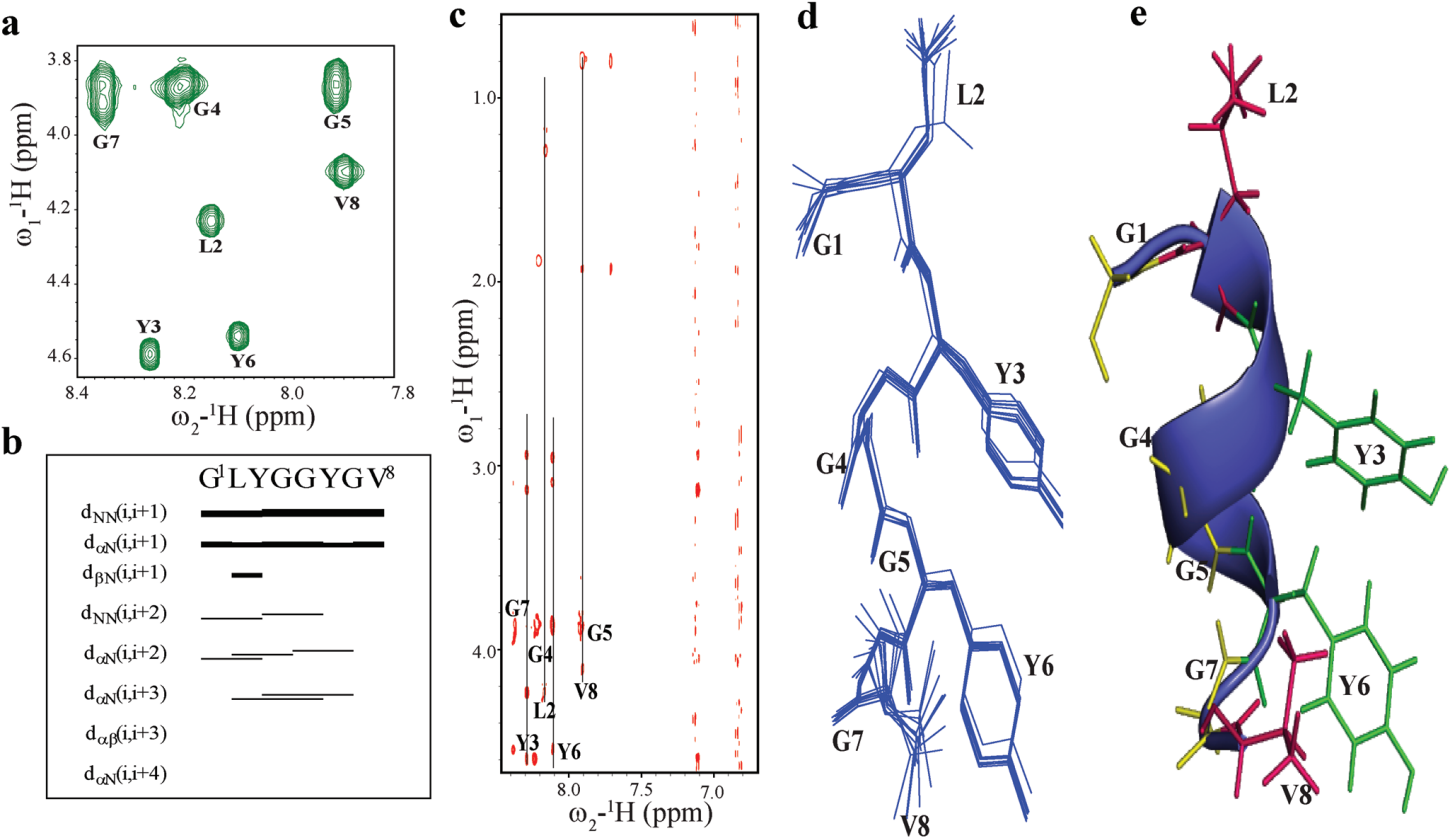
Solution NMR characterization of **GV8** at monomeric concentration (0.5 × 10^−3^ m). a) 2D ^1^H–^1^H TOCSY spectrum of 0.5 × 10^−3^ m **GV8** peptide delineating the individual spins of **GV8** amino acid residues. b) Bar diagram representation of NOE connectivities detected for **GV8** peptide. c) 2D ^1^H–^1^H NOESY spectra displaying weak ring interaction of Y3 and Y6. d) Superimposition of ten lowest energy structures of **GV8** peptide. e) Representative structure of monomeric 3_10_‐helix showing side chain stacking of Y3 and Y6.

In order to obtain the molecular level structure of **GV8**, we analyzed the 3D structure of the peptide in solution using NMR. To maintain the peptide in soluble form, its concentration was kept at 0.5 × 10^−3^ m. 2D ^1^H–^1^H TOCSY (TOtal Correlation SpectroscopY) and ^1^H–^1^H NOESY (Nuclear Overhauser Effect SpectroscopY) spectra showed well‐resolved cross‐peaks assigned to the individual amino acid residues of **GV8** (**Figure** **3**a,c). The ^1^H^α^ chemical shift deviations (CSD)^44^ exhibited negative chemical shifts suggestive of a predominantly helical structure (Figure S6a, Supporting Information). However, precise examination of the ^1^H–^1^H NOESY spectrum revealed the absence of (*i, i+4*) medium range (H^α^–H^N^) NOE connectivities typically observed in α‐helix (Figure 3b). Instead, we only detected (*i, i+3*) H^α^–H^N^ NOEs in addition to the strong (*i, i+1*) H^N^–H^N^ NOEs, suggesting the presence of 3_10_ helix.^45^ Further analysis also revealed the presence of ring proton NOEs between Y3 and Y6 stabilizing the 3_10_ helical structure (Figure 3c). Collectively, these data indicated that the aromatic side chain interactions between Y3 and Y6 may lead the **GV8** peptide to adopt a well‐defined 3_10_ helix. This was also supported by NOEs between aliphatic side chains of L2 and V8 along the helical axis (Figure 3c). The 3D structure of the **GV8** monomeric 3_10_ helix calculated using a total of 39 NOE constraints (**Table** **1**) is shown in Figure 3d,e. When Val8 was mutated to Leu (**GL8**) and Ala (**GA8**), the aromatic interactions between Y3 and Y6 disappeared and both mutated peptides remained in extended conformations (Figure S6c,d, Supporting Information).

**Table 1:** Structural statistics summary of ten lowest energy structures of **GV8** monomer and oligomer obtained from solution state NMR, and **GV8** hydrogel by ssNMR

	**GV8** monomer (solution NMR)	**GV8** oligomer (solution NMR)	**GV8** hydrogel (ssNMR)
Distance restraints			
Intraresidue (|*i−j*| = 0)	9	18	22
Sequential (|*i−j*| = 1)	14	32	27
Medium range (2≤ |*i−j*|≤4)	16	56	0
Long range (|*i−j*|≤ 5)	0	32	10
Total NOE constraints (solution NMR)/dipolar contacts (ssNMR)	39	138	59
Distance restraints violations			
Number of violations	9	51	33
Maximum violation	≤0.5	≤0.5	≤0.5
Average target function value	4.49	34.36	17.37
Deviation from mean structure			
Backbone atoms [Å]	0.52	0.65	1.49
Heavy atoms [Å]	0.92	0.70	1.75
Ramachandran plot for the mean structure			
% residues in the most favorable and additionally allowed regions	100	100	85
% residues in the generously allowed region	0	0	15
% residues in the disallowed region	0	0	0

To monitor gel formation, both 1D proton and 2D ^1^H–^1^H NOESY spectra of 20 × 10^−3^ m **GV8** were recorded during a 4 h period. The peak intensities of the amide protons arising from residual peptides in solution decreased with time (Figure S6b, Supporting Information), implying that an increasing amount of peptide underwent structural rearrangement and were incorporated in the hydrogel.

**Figure 4:**
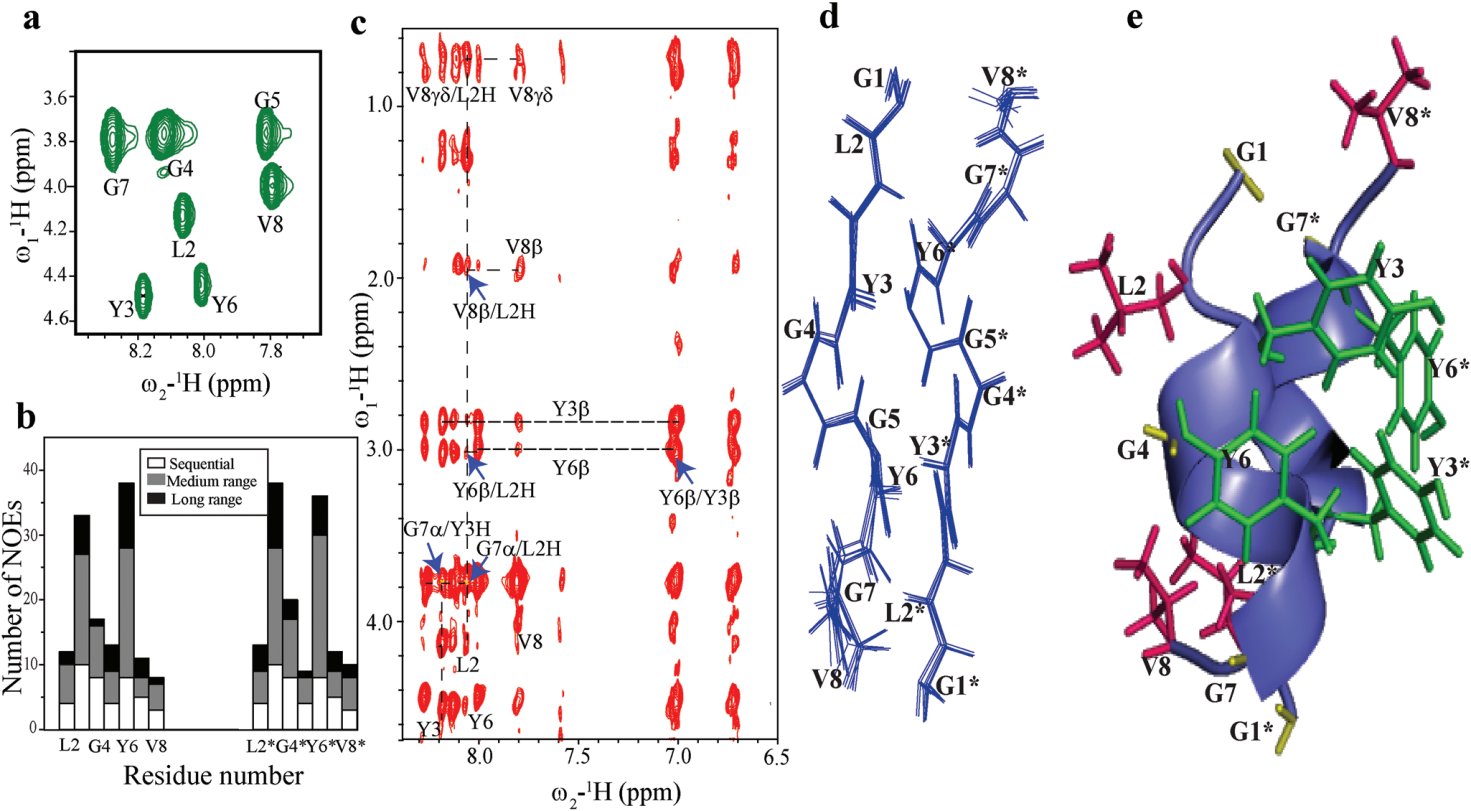
Solution NMR characterization of **GV8** at oligomeric concentration (20 × 10^−3^ m). a) 2D ^1^H–^1^H TOCSY spectrum of 20 × 10^−3^ m **GV8** peptide delineating the individual spins of **GV8** amino acid residues. b) Bar diagram representation of residues that display sequential, medium range, and long‐range NOEs detected at 20 × 10^−3^ m concentration. c) 2D ^1^H–^1^H NOESY spectra displaying long range cross‐strand NOEs and ring proton interactions of Y3 and Y6 (marked with blue arrows and dotted lines). d) Superimposition of ten lowest energy structures of **GV8** peptide at oligomeric 20 × 10^−3^ m concentration. e) Representative structure of dimeric 3_10_‐helix of **GV8** oligomer showing side chain arrangement. Aliphatic residues (L2, V8, L2*, and V8*) are shown in pink color and aromatic residues (Y3, Y6, Y3*, and Y6*) in green.

Analysis of 2D ^1^H–^1^H TOCSY spectra acquired after 20 h demonstrated well resolved cross‐peaks corresponding to individual spins of **GV8** peptide (**Figure** **4**a). 2D ^1^H–^1^H NOESY spectra displayed the (*i, i+3)* NOEs that are fingerprints of a 3_10_‐helix^45^ (Figure 4c). Strikingly, residues at the C‐terminal (G7 and V8) were involved in displaying long range NOEs with residues at N‐terminal (Y3 and L2) (Figure 4c). The H^α^ of G7 interacted with L2 and Y3 residues (blue arrows and dotted lines, Figure 4c) and side chain β protons of V8 were also found to interact with L2 protons. These long‐range NOEs are attributed to cross‐strand NOEs resulting from oligomerization of the **GV8** peptide after 20 h (Figure 4b). The aromatic packing interactions between Y3 and Y6 were also clearly detected owing to the high peptide concentration (Figure 4c).

Using a total of 138 NOE constraints (Table 1), we calculated an ensemble of ten structures for **GV8** composed of dimeric 3_10‐_helical building blocks (adding 5 Gly as a linker between the two monomers). The aromatic residues Y3 and Y6 delineated a higher number of medium and long‐range NOEs (Figure 4b). Superposition of ten lower energy conformers led to root mean square deviations (RMSD) of backbone and heavy chains of 0.65 and 0.70 Å, respectively (Table 1 and Figure 4d). 3D structure calculation revealed that the hydrophobic face of the dimeric 3_10_‐helix is composed of π‐stacking interactions between Y3 and Y6, while the exposed side of the dimeric helix is made up of aliphatic side chains L2 and V8 (Figure 4d,e). Procheck analysis of the 3D structure revealed that all residues resided in the sterically allowed regions (Table 1).

### 2.6 Amide Temperature Coefficient and H/D Exchange NMR Studies

The role of hydrogen bonds in stabilizing the 3_10_‐helix was studied by calculating the protection factors from H/D exchange as well as the amide proton temperature coefficients (Δδ_NH_/Δ*T*) at various temperatures. A series of 2D ^1^H–^1^H TOCSY spectra were recorded every 30 min for the 0.5 and 20 × 10^−3^ m **GV8** peptide dissolved in D_2_O. All Gly residues for both the monomer (0.5 × 10^−3^ m) and the oligomer (20 × 10^−3^ m) concentrations displayed protection factor of 60–80, supporting a significant H/D exchange protection inside the core of the 3_10_‐helical structure (Figure S7a, Supporting Information). The protection factor of Y3 and Y6 increased with the peptide concentration, indicating enhanced aromatic interactions for the oligomeric form (Figure S7a, Supporting Information). Comparably, the amide proton temperature coefficients of all **GV8** residues at both monomer and oligomer concentrations exhibited values more positive than −4.6 ppb/K.^46^ The Gly residues also exhibited more positive values in line with their higher protection factor values (Figure S7b, Supporting Information).

### 2.7 Solid State NMR

**Figure 5:**
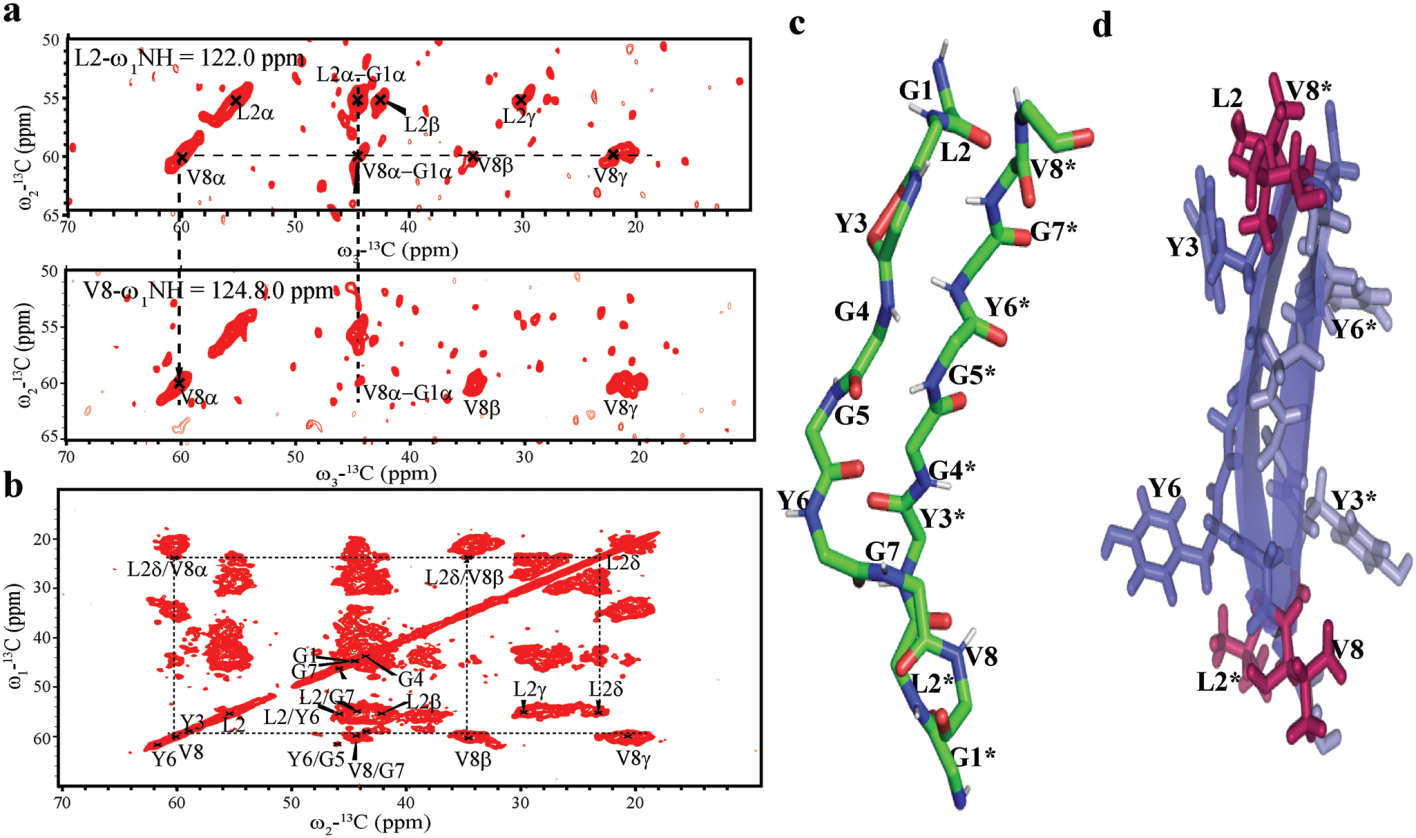
Solid NMR characterization of ^13^C–^15^N labeled **GV8** hydrogel. a) Strip plots of L2 and V8, 3D NCACX spectra of ^13^C–^15^N labeled **GV8** peptide hydrogel displaying long‐range contact of residues. b) 2D ^13^C–^13^C DARR spectra with contact time of 50 ms showing long‐range dipolar contacts between L2 and V8 side chains (β, δ, γ). c) Representative structure of dimeric extended conformation of **GV8** hydrogel. d) Side chain disposition representation of antiparallel β‐sheets of **GV8** hydrogel displaying interchain connectivity between L2 and V8 residues (L2/V8* and L2*/V8).

NMR characterizations of the gel state were conducted by ssNMR under magic angle spinning (MAS) conditions. All amino acids of **GV8** hydrogel prepared with uniformly labeled ^13^C and ^15^N peptide were unambiguously assigned using the sequential walking method of 3D NCACX, NCOCX, and CANcoCX spectra (**Figure** **5**a). Analysis of the 2D ^13^C–^13^C DARR spectra acquired at 50 ms contact time revealed long range dipolar contacts between L2 and V8 side chains (Figure 5a,b). The Y3/Y6 ring packing interactions that were detected in oligomeric solutions of **GV8** were no longer present in the hydrogel state.

We then calculated the dimeric conformation of **GV8** in the hydrogel state using intraresidue and sequential dipolar constraints (Experimental Section). An overview of ten lowest energy structures resulted in RMSD value of 1.49 Å for backbone atoms and 1.75 Å for heavy side chain atoms (Table 1). The lowest energy structure revealed that the **GV8** hydrogel comprised of extended antiparallel β‐sheets (Figure 5c). The absence of Tyr ring packing interactions strongly suggests that during gelation, Tyr side chains (Y3 and Y6) rearranged to be exposed to solvent, and that at the same time stronger hydrophobic interactions between L2, V8, L2*, and V8* stabilized the antiparallel β‐strand conformation of the **GV8** hydrogel (Figure 5d). Extended 3_10_‐helices have been shown to act as intermediate seeds for the formation of amyloid (β‐sheet rich) aggregates,^47^ but to the best of our knowledge it has previously not been reported to induce hydrogel formation. Furthermore, none of the Val8‐mutated peptides (Figure S1, Supporting Information) gelled under the same conditions and neither control peptides **GL8** nor **GA8** formed 3_10_‐helices in solution (Figure S6c,d, Supporting Information) hence corroborating that 3_10_‐helix is a critical transient conformation leading to gelation of **GV8**. Val8 at the C‐terminus therefore plays a crucial role in stabilizing the 3_10_‐helix structural intermediate through an intrachain hydrophobic interaction, which is not achieved with Ala or Leu residues.

### 2.8 WAXS of Extruded GV8 Hydrogel and MD Simulations

Confirmation of β‐sheet presence in the gel was gained by performing WAXS measurements in both the wet and dry states. In the wet state (Figure 2b) a peak at *q* = 5.44 nm^−1^ and a shoulder at 13.3 nm^−1^ were observed, corresponding to distances of 1.16 nm and 4.7 Å, respectively. These features are the hallmark of β‐sheet rich amyloid fibrils, with 1.16 nm corresponding to the inter‐β‐sheet spacing and 4.7 Å to the interstrand distance of β‐sheets. Interestingly, both features were greatly enhanced upon drying of the gel: the peak at 1.16 nm shifted to 1.09 nm due to dehydration, whereas the 4.7 Å peak now became the dominant scattering peak of the dried gel. The emergence of additional correlations is highlighted by a deconvolution of the intensity profile with Laurentian curves as shown in Figure 2c.

**Figure 6:**
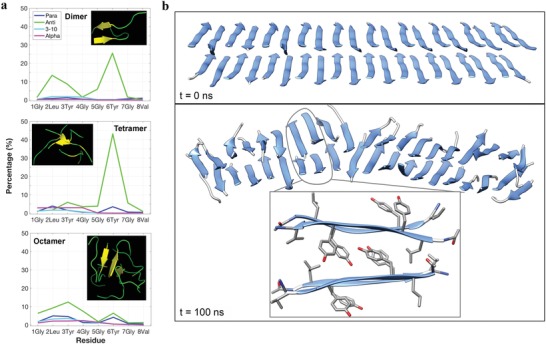
MD simulations of **GV8** conformation and oligomeric self‐assembly. a) Secondary structure distribution of each residue in dimer, tetramer, and octamer of **GV8** at 300K for 200 ns. b) Initial and final structures of a 40‐mer model of the **GV8** peptide with β‐sheets structures shown in blue. The inset in the bottom panel shows the representative structure in the model, whereby the π–π stacking and hydrophobic interactions mainly contribute to intersheet association.

To further assess the conformation propensity of **GV8**, we conducted MD simulations on both oligomeric and 40‐mer constructs. These simulations predicted that oligomers of **GV8** prefer the antiparallel β‐sheet conformation, especially the Leu2, Tyr3, and Tyr6 residues (**Figure** **6**a), whereas 3_10_‐helices were not stable thus resulting in a very low concentration of 3_10_‐helices after 200 ns simulations. These results indicate that antiparallel β‐sheets constituted the most stable structure at equilibrium. It is important to emphasize that the peptide concentration during MD simulations is much higher than in the soluble form and more representative of the gel state. Therefore, the very low concentration of 3_10_‐helices at equilibrium is in line with the conformational transition detected in the gel by NMR. Based on solid‐state NMR and WAXS data in the gel state, we then conducted simulations on a 40‐mer antiparallel β‐sheet construct. The 100 ns simulation indicated a very high stability of antiparallel β‐sheets, in agreement with the WAXS measurements. Furthermore, the simulations indicated that β‐strands were stabilized by intersheet π–π stacking of Tyr residues (Figure 6b). This result corroborates the molecular‐level structure of **GV8** by solid‐state NMR, which indicated that Tyr side‐chains in the gel state pointed out perpendicular to the strand direction, making them available to engage in intersheet interactions as predicted by the simulations. In addition, in‐register antiparallel β‐sheets were observed for both ssNMR and MD simulations, which we postulate is due to the arrangement of the Tyr side‐chains that leads to the least sterically‐hindered conformation.

## 3 Conclusions

**GV8** is an eight amino acid long peptide repeat from suckerin‐19—the most abundant protein forming the load‐bearing squid sucker ring teeth—that forms stiff hydrogels in water with tunable elastic modulus. Using CD, FTIR, and solution NMR spectroscopy, we have determined that **GV8** self‐assembles into unusual 3_10_ monomeric helices at low peptide concentration, which are intramolecularly stabilized by π–π stacking aromatic interactions between Y3 and Y6 residues, as well by the aliphatic side chains L2 and V8. As the concentration increases, **GV8** dimerizes into antiparallel 3_10_ helices driven by π‐stacking interactions between Tyr residues Y3, Y6, Y3*, and Y6*. In the gel state, ssNMR and WAXS measurements indicate that **GV8** is made of antiparallel β‐sheets, inferring that gelation proceeds by a 3_10_‐helix to β‐sheet conformational rearrangement. This mechanism is starkly different from previous reports on fibrous peptide‐based hydrogels. During this conformational transition, Tyr side‐chains reorient perpendicular to the chain direction according to both ssNMR and MD simulations, allowing to mediate intersheet interactions. Peptide‐based hydrogels with water gelation and the ability to tune the stiffness 25‐fold simply by increasing the peptide concentration may find notable opportunities for biomedical applications, such as tissue engineering, encapsulation of therapeutics, soft tissue adhesives, or matrix for stem cell differentiation.

## 4 Experimental Section

*Materials*: Ac‐GLYGGYGV‐NH_2_ peptide and Ac‐GLYGGYGX‐NH_2_ peptides (where X = V, L, A, F, S, K, and I) were purchased from GL Biochem (Shanghai) Ltd. Peptides were checked to be >98% purity via trace HPLC and LC/MS prior to use. All of the peptides were acetylated at the N‐terminal and amidated at the C‐terminal to prevent end‐to‐end charge interactions. ^13^C–^15^N uniformly labeled **GV8** crude peptide purchased from Cambridge Isotopes was purified to >95% purity via HPLC and checked with LC/MS prior to use.

*UV–vis Spectroscopy*: The peptides were dissolved in DI water at the respective concentrations and 100 µL was aliquoted into each well of a 96‐well microtiter plate, with a minimum of three wells per condition. UV–vis absorbance measurements at 550 nm were recorded on a Tecan infinite M200 Pro microplate reader at intervals of 30 min for the first 16 h and subsequently at increased time intervals.

*Peptide Hydrogel*: **GV8** peptide was dissolved in DI water at the desired concentration (between 10 and 20 × 10^−3^ m) and incubated at ambient temperature for at least 12 h.

*CD Spectroscopy*: **GV8** peptide was dissolved at 20 × 10^−3^ m concentration in DI water and spectra were collected using a 0.2 mm path length quartz cuvette. Data acquisition was performed using AVIV 420 Circular Dichroism (New Jersey, USA) spectrometer. A quartz sandwich cuvette with optical path length of 0.2 mm was used for all data collection and the edges of the cuvette were sealed with parafilm to prevent loss of liquid. Data were acquired over a wavelength range of 190–260 nm and acquisition parameters were 0.5 nm wavelength steps with an averaging time of 0.1 s, 1.00 nm bandwidth, and readings were averaged over three scans. Obtained spectra were smoothed at 12 pts via adjacent‐averaging method (ensuring that no visible existing peaks were removed or artefacts introduced) and plotted via OriginPro 9.1.

*FTIR Spectroscopy*: ATR‐FTIR spectroscopy of lyophilized **GV8** samples were performed on a Bruker Vertex 70 (Massachusetts, USA) equipped with a PIKE Technologies MIRacle attenuated total reflection (ATR) ZnSe‐Diamond 3‐reflection accessory and a LN_2_ cooled MCT detector. Scans were obtained at ambient temperature over the range of 4000–750 cm^−1^ with a resolution of 2 cm^−1^, averaged over 128 scans. **GV8** peptide solutions were prepared at 20 × 10^−3^ m concentration in separate vials of 20 µL and snap freezed by dipping the vials in liquid N_2_ for 5 min at the stipulated time points and lyophilized immediately. All spectra processing were performed on OPUS 6.5, and processed in the sequence of water vapor subtraction, baseline correction, then normalized using amide I band. Amide I band was deconvoluted by secondary derivation, with peak fitting performed using 100% Gaussian curves with individual FWHM kept relatively consistent. The deconvoluted peaks were assigned to β‐sheet, unordered, helix and turns or 3_10_ structures.^40^, ^41^, ^48^, ^49^

*Cryo‐EM*: **GV8** peptide was dissolved at a concentration of 20 × 10^−3^ m and incubated for 3 h. Copper grids with Ultrathin C Film on Lacey Carbon support film was plasma‐treated with JEOL DATUM HDT‐400 for 300 s to increase hydrophilicity of grid surfaces to allow aqueous samples to adhere and spread. Vitrified samples were prepared using Gatan Cryoplunge^TM^3 (Cp3). 4 µL of sample was pipetted onto each plasma‐treated copper grid and blotted for 5 s followed by vitrification in liquid ethane at −180 °C. All images were taken in bright‐field mode with objective aperture inserted. Imaging was carried out with energy filtered Carl Zeiss TEM, LIBRA 120 with in‐column Omega spectrometer and operated an acceleration voltage of 120 kV and the sample temperature was maintained below −180 °C during imaging.

*SEM*: Peptide hydrogel was snap freezed by dipping into liquid N_2_ for at least 5 min. The frozen hydrogel was then cryo‐fractured with tweezers to expose the porous cross‐section and the fractured surfaces were placed face‐up on carbon tape and lyophilized immediately. Samples were Platinum‐coated below 5 Pa, at 20 mA for 30 s and imaging was performed using JEOL JSM‐FESEM 7600F (Massachusetts, USA), at SEI‐mode, 5 kV, and 92 µA emission current.

*AFM*: 10 µL of **GV8** peptide at 20 × 10^−3^ m concentration was deposited onto freshly cleaved mica and air‐dried overnight. AFM images were obtained on Asylum Cypher S AFM (Oxfordshire, UK) in tapping mode using Nanoworld NCSTR silicon nitride soft‐tip cantilevers (*R*_f_ = 160 kHz, *k* = 7.4 N m^−1^). All images were flattened to remove background curvature using Igor Pro software and no further image processing was carried out.

*Rheology*: Rheological measurements were performed at ambient temperature on Anton Paar MCR501 rheometer with a parallel plate PP10 geometry. Five different concentrations (10, 12, 15, 18, and 20 × 10^−3^ m) of **GV8** hydrogels were prepared in DI water, each pipetted into 1 mL syringes with nozzles removed, then left overnight to gelate. The hydrogel was extruded from the syringes and cut to 1–2 mm thick slices with a sterile blade and placed on the rheometer plate for measurements. Strain sweeps were first conducted at constant frequency of 1 Hz, from 0.1% to 10 % strain to identify the linear viscoelastic region and 0.25% strain was selected for subsequent frequency sweeps that were conducted from 0.01 to 100 Hz. All measurements were triplicated with repeated measurements performed on fresh samples.

*NMR*: All solution state NMR experiments were carried out on a Bruker 700 MHz spectrometer equipped with a cryoprobe. NMR data were processed with TOPSPIN (Bruker), then analyzed using Sparky^50^ programs. 0.5 or 20 × 10^−3^ m **GV8** peptide was dissolved in water, pH 6.8 with 10% D_2_O for deuterium lock and DSS for signal reference. 2D ^1^H–^1^H TOCSY and NOESY spectra were acquired with 80 and 200 ms mixing times, respectively. In order to monitor hydrogel formation, 1D ^1^H spectrum and a series of 2D ^1^H–^1^H NOESY spectra were recorded every 4 h for 20 h using the 20 × 10^−3^ m peptide solution. For H/D exchange experiments, 0.5 and 20 × 10^−3^ m peptide samples were dissolved in 100% D_2_O and 2D ^1^H–^1^H TOCSY spectra were recorded at 30 min intervals. The extrinsic exchange rates were obtained by fitting the peak intensity versus time to a single‐exponential decay equation. The protection factor were calculated as the ratio of intrinsic exchange rates (calculated from SPHERE^51^) to the extrinsic exchange rates. A protection factor above 30 is indicative of stable hydrogen bonds, while values between 10 and 30 indicate an intermediate range of hydrogen bond strength.^52^ Amide temperature coefficients were also determined by recording 1D ^1^H spectra of 0.5 and 20 × 10^−3^ m **GV8** peptide at 298, 303, 308, and 313 K. Amide proton chemical shift deviations were fitted linearly against temperature and the temperature coefficients were calculated as *σδ*HN/Δ*T* (ppb K^−1^).

Solid state NMR experiments were carried out on a Bruker 600 MHz spectrometer equipped with a 1.7 mm MAS probe. The MAS spinning frequency was 13333 Hz. 20 × 10^−3^ m of ^13^C–^15^N labeled **GV8** peptide was dissolved in water, pH 6.8 and allowed to incubate overnight for hydrogel formation. Sample was loaded in a 1.7 mm thin wall zirconia rotor (Bruker) manually and the rotor was spun at 70 000 rpm for 30 min by ultracentrifugation (Beckman Proteomelab XL, IN, USA). 2D ^13^C–^13^C DARR spectra were recorded over contact times ranging from 50 to 400 ms. 3D NCACX, NCOCX, and CANcoCX experiments were also recorded with 50 ms contact time.

*NMR Structure Calculation*: The structure calculations were carried out using the CYANA 2.1 program. The monomeric conformation of **GV8** was calculated using the intensities of ^1^H–^1^H NOE cross peaks that were classified as strong, medium, and weak and translated to upper bound distance limits of 2.5, 3.5, and 5.0 Å. The dihedral Φ and Ψ angles were constrained between −120° to −30° and −120° to 120° as suggested in the CYANA program files. Out of the 100 structures generated, the ten lowest energy structures were used for more analysis. The dimer structures of **GV8** were also calculated using the same constraints as monomeric structure calculation. The two monomeric units were linked by five glycine linkers. The structure of hydrogel was calculated using ^13^C–^13^C dipolar contacts derived from solid state NMR spectra. All of the conformations were validated using PROCHECK.^53^ For structure calculation from ssNMR, a total of 47 intraresidue and sequential dipolar constraints were used. The long‐range dipolar contacts included in the structure calculation were cross‐strand contacts used to generate a dimeric conformation.

*SAXS and WAXS*: SAXS and WAXS experiments were performed using Rigaku MicroMax‐002+ equipped with a microfocused beam (40 W, 45 kV, 0.88 mA) with the λ_Cu Kα_ = 0.15418 nm radiation collimated by three pinhole collimators (0.4, 0.3, and 0.8 mm). The SAXS and WAXS intensities were collected by a two‐dimensional Triton‐200 gas‐filled X‐ray detector (20 cm diameter, 200 µm resolution) and a 2D Fujifilm BAS‐MS 2025 imaging plate system (15.2 × 15.2 cm^2^, 50 µm resolution), respectively. An effective scattering vector range of 0.05 nm^−1^ < *q* < 25 nm^−1^ was obtained, where *q* is the scattering wave vector defined as *q* = 4π sin θ/λ_Cu Kα_ with a scattering angle of 2θ.

*Hamiltonian‐Replica Exchange Molecular Dynamics (H‐REMD) Simulations*: H‐REMD simulations^54^ were performed for the dimer, tetramer, and octamer of the Ac‐GLYGGYGV‐NH_2_ peptide for 200 ns each. The CHARMM 36 mm force field parameters^55^ were applied to peptides, and the dimer, tetramer, and octamer were put in a cubic box with TIP3P waters^56^ and 0.15 m NaCl. The minimum distance between the peptides and the box edge was larger than 1.5 nm. The dimer, tetramer, and octamer systems have 8, 12, and 16 replicas from 300 to 600 K, respectively, and each was simulated for 200 ns. The trajectories were saved every 2 ps.

*Conventional MD Simulations*: Conventional MD simulations were performed for the 40‐mer two‐layer antiparallel β‐sheet model for 100 ns using the AMBER 16 software^57^ together with the AMBER14SB force field. SHAKE algorithm^58^ was used to constrain all bonds involving hydrogens and electrostatic interactions were treated by the particle mesh Ewald sum method^59^ with a 8 Å cutoff for nonbonded interactions in direct space. The model was solvated in a rectangular box filled with TIP3P waters,^56^ with an at least 1.0 nm distance between the peptides and the box edge. The whole system was first energy‐minimized, with a series of position restraints on the solute (all heavy atoms, backbone atoms, and Cα atoms). The simulation was continued for 100 ns at 1 bar and 298.15 K.

## Conflict of Interest

The authors declare no conflict of interest.

## Supporting information

Supplementary

Supplementary

## References

[1] Y. Loo , Y. C. Wong , E. Z. Cai , C. H. Ang , A. Raju , A. Lakshmanan , A. G. Koh , H. J. Zhou , T. C. Lim , S. M. Moochhala , C. A. E. Hauser , Biomaterials 2014, 35, 4805. 24636214 10.1016/j.biomaterials.2014.02.047

[2] N. C. Carrejo , A. N. Moore , T. L. L. Silva , D. G. Leach , I. C. Li , D. R. Walker , J. D. Hartgerink , ACS Biomater. Sci. Eng. 2018, 4, 1386. 29687080 10.1021/acsbiomaterials.8b00031 PMC5909404

[3] Y. Nagai , H. Yokoi , K. Kaihara , K. Naruse , Biomaterials 2012, 33, 1044. 22056753 10.1016/j.biomaterials.2011.10.049

[4] J. Y. Li , D. J. Mooney , Nat. Rev. Mater. 2016, 1, 16071. 29657852 10.1038/natrevmats.2016.71 PMC5898614

[5] B. Trappmann , J. E. Gautrot , J. T. Connelly , D. G. T. Strange , Y. Li , M. L. Oyen , M. A. C. Stuart , H. Boehm , B. J. Li , V. Vogel , J. P. Spatz , F. M. Watt , W. T. S. Huck , Nat. Mater. 2012, 11, 642. 22635042 10.1038/nmat3339

[6] N. Stephanopoulos , J. H. Ortony , S. I. Stupp , Acta Mater. 2013, 61, 912. 23457423 10.1016/j.actamat.2012.10.046 PMC3580867

[7] H. G. Cui , M. J. Webber , S. I. Stupp , Biopolymers 2010, 94, 1. 20091874 10.1002/bip.21328 PMC2921868

[8] S. F. Hedegaard , M. S. Derbas , T. K. Lind , M. R. Kasimova , M. V. Christensen , M. H. Michaelsen , R. A. Campbell , L. Jorgensen , H. Franzyk , M. Cardenas , H. M. Nielsen , Sci. Rep. 2018, 8, 6327. 29679078 10.1038/s41598-018-24154-z PMC5910404

[9] M. A. Gonzalez , J. R. Simon , A. Ghoorchian , Z. Scholl , S. T. Lin , M. Rubinstein , P. Marszalek , A. Chilkoti , G. P. Lopez , X. H. Zhao , Adv. Mater. 2017, 29, 1604743. 10.1002/adma.201604743 28060425

[10] C. Q. Yan , D. J. Pochan , Chem. Soc. Rev. 2010, 39, 3528. 20422104 10.1039/b919449p PMC3104857

[11] M. A. Khalily , M. Goktas , M. O. Guler , Org. Biomol. Chem. 2015, 13, 1983. 25566850 10.1039/c4ob02217c

[12] Y. H. Loo , A. Lakshmanan , M. Ni , L. L. Toh , S. Wang , C. A. E. Hauser , Nano Lett. 2015, 15, 6919. 26214046 10.1021/acs.nanolett.5b02859

[13] E. F. Banwell , E. S. Abelardo , D. J. Adams , M. A. Birchall , A. Corrigan , A. M. Donald , M. Kirkland , L. C. Serpell , M. F. Butler , D. N. Woolfson , Nat. Mater. 2009, 8, 596. 19543314 10.1038/nmat2479 PMC2869032

[14] J. K. Sahoo , M. A. VandenBerg , M. J. Webber , Adv. Drug Delivery Rev. 2018, 127, 185. 10.1016/j.addr.2017.11.005 29128515

[15] S. F. Souza , S. Kogikoski , E. R. Silva , W. A. Alves , J. Braz. Chem. Soc. 2017, 28, 1619.

[16] P. A. Guerette , S. Hoon , D. W. Ding , S. Amini , A. Masic , V. Ravi , B. Venkatesh , J. C. Weaver , A. Miserez , ACS Nano 2014, 8, 7170. 24911543 10.1021/nn502149u

[17] S. H. Hiew , P. A. Guerette , O. J. Zvarec , M. Phillips , F. Zhou , H. Su , K. Pervushin , B. P. Orner , A. Miserez , Acta Biomater. 2016, 46, 41. 27693688 10.1016/j.actbio.2016.09.040

[18] S. H. Hiew , A. Miserez , ACS Biomater. Sci. Eng. 2016, 3, 680. 10.1021/acsbiomaterials.6b00284 33440495

[19] A. Kumar , H. Mohanram , K. W. Kong , R. Goh , S. Hoon , J. Lescar , A. Miserez , Biomater. Sci. 2018, 21, 401. 10.1039/c8bm00556g 30042992

[20] H. G. Zhao , L. Ma , J. Zhou , Z. W. Mao , C. Y. Gao , J. C. Shen , Biomed. Mater. 2008, 3, 015001. 18458488 10.1088/1748-6041/3/1/015001

[21] C. J. Newcomb , T. J. Moyer , S. S. Lee , S. I. Stupp , Curr. Opin. Colloid Interface Sci. 2012, 17, 350. 23204913 10.1016/j.cocis.2012.09.004 PMC3510006

[22] F. C. Mackintosh , J. Kas , P. A. Janmey , Phys. Rev. Lett. 1995, 75, 4425. 10059905 10.1103/PhysRevLett.75.4425

[23] C. Storm , J. J. Pastore , F. C. MacKintosh , T. C. Lubensky , P. A. Janmey , Nature 2005, 435, 191. 15889088 10.1038/nature03521

[24] Y. P. Cao , S. Bolisetty , J. Adamcik , R. Mezzenga , Phys. Rev. Lett. 2018, 120, 158103. 29756901 10.1103/PhysRevLett.120.158103

[25] M. L. Gardel , J. H. Shin , F. C. MacKintosh , L. Mahadevan , P. Matsudaira , D. A. Weitz , Science 2004, 304, 1301. 15166374 10.1126/science.1095087

[26] R. Mezzenga , P. Fischer , Rep. Prog. Phys. 2013, 76, 046601. 23455715 10.1088/0034-4885/76/4/046601

[27] M. A. Greenfield , J. R. Hoffman , M. O. de la Cruz , S. I. Stupp , Langmuir 2010, 26, 3641. 19817454 10.1021/la9030969

[28] B. Ozbas , J. Kretsinger , K. Rajagopal , J. P. Schneider , D. J. Pochan , Macromolecules 2004, 37, 7331.

[29] A. Bertolani , L. Pirrie , L. Stefan , N. Houbenov , J. S. Haataja , L. Catalano , G. Terraneo , G. Giancane , L. Valli , R. Milani , O. Ikkala , G. Resnati , P. Metrangolo , Nat. Commun. 2015, 6, 7574. 26123690 10.1038/ncomms8574 PMC4491812

[30] D. J. Adams , M. F. Butler , W. J. Frith , M. Kirkland , L. Mullen , P. Sanderson , Soft Matter 2009, 5, 1856.

[31] W. Y. Seow , G. Salgado , E. B. Lane , C. A. E. Hauser , Sci. Rep. 2016, 6, 32670. 27600999 10.1038/srep32670 PMC5013444

[32] D. E. Discher , P. Janmey , Y. L. Wang , Science 2005, 310, 1139. 16293750 10.1126/science.1116995

[33] A. J. Engler , S. Sen , H. L. Sweeney , D. E. Discher , Cell 2006, 126, 677. 16923388 10.1016/j.cell.2006.06.044

[34] M. Ahearne , Interface Focus 2014, 4, 20130038. 24748951 10.1098/rsfs.2013.0038 PMC3982445

[35] I. B. Grishina , R. W. Woody , Faraday Discuss. 1994, 99, 245. 10.1039/fd9949900245 7549540

[36] M. A. Khan , C. Neale , C. Michaux , R. Pomes , G. G. Prive , R. W. Woody , R. E. Bishop , Biochemistry 2007, 46, 4565. 17375935 10.1021/bi602526k PMC5007129

[37] A. G. Cochran , N. J. Skelton , M. A. Starovasnik , Proc. Natl. Acad. Sci. USA 2001, 98, 5578. 11331745 10.1073/pnas.091100898 PMC33255

[38] L. Wu , D. McElheny , T. Takekiyo , T. A. Keiderling , Biochemistry 2010, 49, 4705. 20423111 10.1021/bi100491s

[39] R. W. Woody , Biopolymers 1978, 17, 1451.

[40] J. Kong , S. Yu , Acta Biochim. Biophys. Sin. 2007, 39, 549. 17687489 10.1111/j.1745-7270.2007.00320.x

[41] H. Y. Yang , S. N. Yang , J. L. Kong , A. C. Dong , S. N. Yu , Nat. Protoc. 2015, 10, 382. 25654756 10.1038/nprot.2015.024

[42] R. Armen , D. O. V. Alonso , V. Daggett , Protein Sci. 2003, 12, 1145. 12761385 10.1110/ps.0240103 PMC2323891

[43] A. G. de Brevern , Sci. Rep. 2016, 6, 33191. 27627963 10.1038/srep33191 PMC5024104

[44] D. S. Wishart , B. D. Sykes , F. M. Richards , Biochemistry 1992, 31, 1647. 1737021 10.1021/bi00121a010

[45] G. L. Millhauser , C. J. Stenland , P. Hanson , K. A. Bolin , F. J. M. van de Ven , J. Mol. Biol. 1997, 267, 963. 9135124 10.1006/jmbi.1997.0923

[46] J. O. Tornasz Cierpicki , J. Biomol. NMR 2001, 21, 249. 11775741 10.1023/a:1012911329730

[47] Y. Singh , P. C. Sharpe , H. N. Hoang , A. J. Lucke , A. W. McDowall , S. P. Bottomley , D. P. Fairlie , Chem. ‐ Eur. J. 2011, 17, 151. 21207612 10.1002/chem.201002500

[48] D. Wilson , R. Valluzzi , D. Kaplan , Biophys. J. 2000, 78, 2690. 10777765 10.1016/S0006-3495(00)76813-5 PMC1300858

[49] E. Vass , M. Hollosi , F. Besson , R. Buchet , Chem. Rev. 2003, 103, 1917. 12744696 10.1021/cr000100n

[50] W. Lee , M. Tonelli , J. L. Markley , Bioinformatics 2015, 31, 1325. 25505092 10.1093/bioinformatics/btu830 PMC4393527

[51] Y. Bai , J. S. Milne , L. Mayne , S. W. Englander , Proteins: Struct., Funct., Genet. 1993, 17, 75. 8234246 10.1002/prot.340170110 PMC3438223

[52] T. M. K. Kuwata , H. Cheng , K. Nagayama , T. L. James , H. Roder , Proc. Natl. Acad. Sci. USA 2003, 100, 14790. 14657385 10.1073/pnas.2433563100 PMC299804

[53] R. A. Laskowski , M. W. MacArthur , D. S. Moss , J. M. Thornton , J. Appl. Crystallogr. 1993, 26, 283.

[54] G. Bussi , Mol. Phys. 2014, 112, 379.

[55] J. Huang , A. D. MacKerell Jr ., J. Comput. Chem. 2013, 34, 2135. 23832629 10.1002/jcc.23354 PMC3800559

[56] W. Jorgensen , J. Chandrasekhar , J. Madura , R. Impey , M. Klein , J. Chem. Phys. 1983, 79, 926.

[57] D. A. Case , T. E. Cheatham , T. Darden , H. Gohlke , R. Luo , K. M. Merz , A. Onufriev , C. Simmerling , B. Wang , R. J. Woods , J. Comput. Chem. 2005, 26, 1668. 16200636 10.1002/jcc.20290 PMC1989667

[58] J. P. Ryckaert , G. Ciccotti , H. J. C. Berendsen , J. Comput. Phys. 1977, 23, 327.

[59] U. Essmann , L. Perera , M. L. Berkowitz , T. Darden , H. Lee , L. G. Pedersen , J. Chem. Phys. 1995, 103, 8577.

