# Supplementary material for: A Short Peptide Hydrogel with High Stiffness Induced by 310‐Helices to β‐Sheet Transition in Water
Source: Adv Sci (Weinh). 2019 Sep 10;6(21):1901173. doi: 10.1002/advs.201901173 (PMC6839752; doi:10.1002/advs.201901173)
Supplement: Supplementary file 1 — Supplementary [file ADVS-6-1901173-s002.pdf]

Copyright WILEY-VCH Verlag GmbH & Co. KGaA, 69469 Weinheim, Germany, 2019.

Supporting Information

for *Adv. Sci.,* DOI: 10.1002/advs.201901173

A Short Peptide Hydrogel with High Stiffness Induced by 3_{10}-Helices to β-Sheet Transition in Water

*Shu Hui Hiew, Harini Mohanram, Lulu Ning, Jingjing Guo, Antoni Sánchez-Ferrer, Xiangyan Shi, Konstantin Pervushin, Yuguang Mu, Raffaele Mezzenga, and Ali Miserez**

A Short Peptide Hydrogel with High Stiffness Induced by 3_{10}-Helices to β-Sheet Transition in Water

Shu Hui Hiew, Harini Mohanram, Lulu Ning, Jingjing Guo, Antoni Sánchez-Ferrer, Xiangyan Shi, Konstantin Pervushin, Yuguang Mu, Raffaele Mezzenga, and Ali Miserez[*]

Dr. S.H. Hiew, Dr.H. Mohanram, Prof.A. Miserez.

Center for Biomimetic Sensor Science,

School of Materials Science and Engineering,

Nanyang Technological University,

Singapore 639798, Singapore.

E-mail: ali.miserez@ntu.edu.sg.

Dr.L. Ning, J. Guo, Dr. X.Shi, Prof. K. Pervushin, Prof. Y. Mu, Prof. A. Miserez.

School of Biological Sciences,

Nanyang Technological University,

Singapore 637551, Singapore.

Dr. A. Sánchez-Ferrer, Prof. R. Mezzenga.

Department of Health Sciences & Technology,

ETH Zurich,

Switzerland CH-8092, CH-8093.

Key-words: suckerin, peptide hydrogel, β-Sheet Transition, NMR spectroscopy, MD simulations.

Supplementary Figures

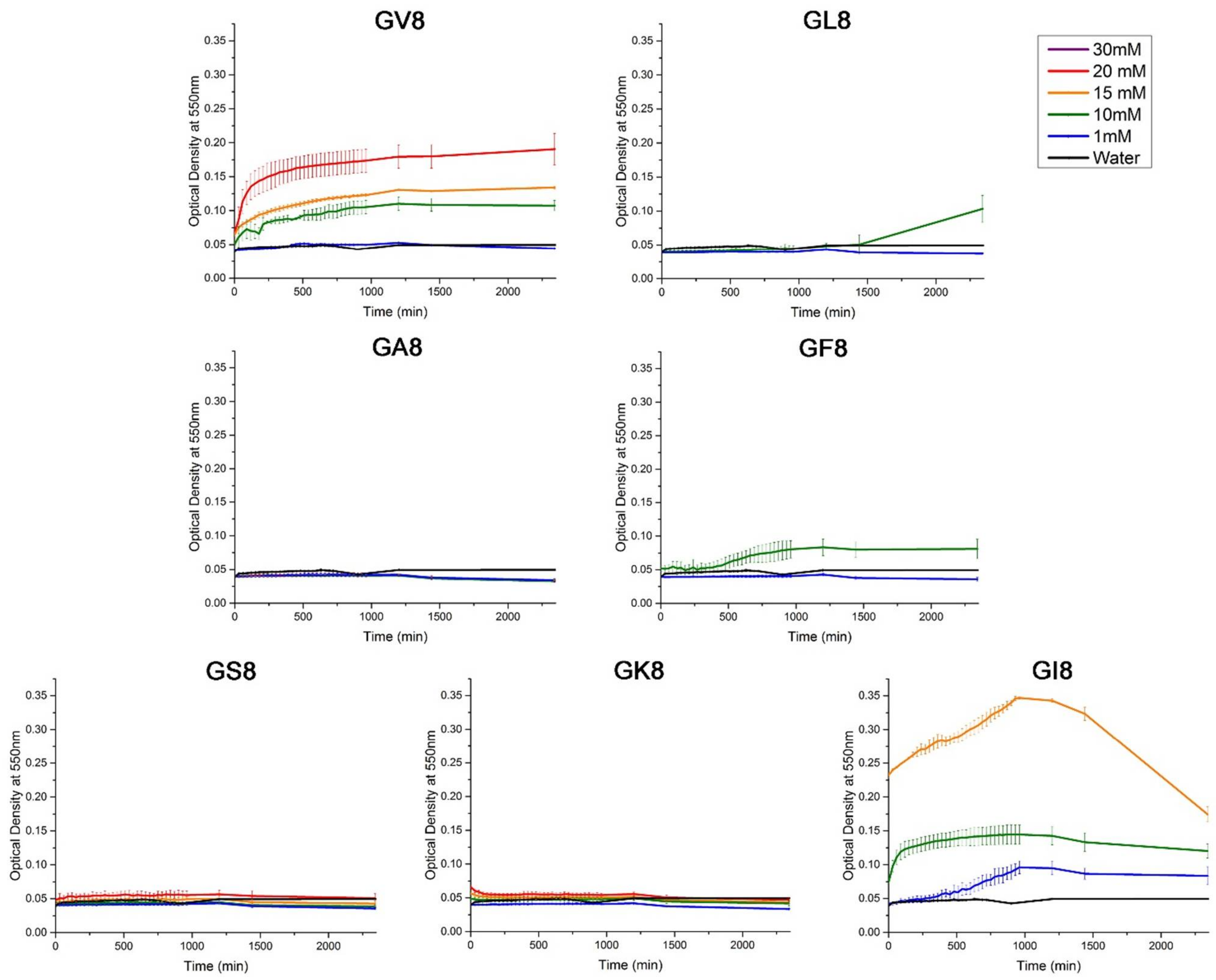

Figure S1. Gelation kinetics observed via optical density measurements of **GX8** peptide solutions**.** Peptides **GV8**, **GL8**, **GA8**, **GF8**, **GS8**, **GK8** and **GI8** were incubated in DI water at various concentrations over a period of 40 h and observed for gelation via their absorbance at 550 nm. Some sequences have higher solubilities than the others.

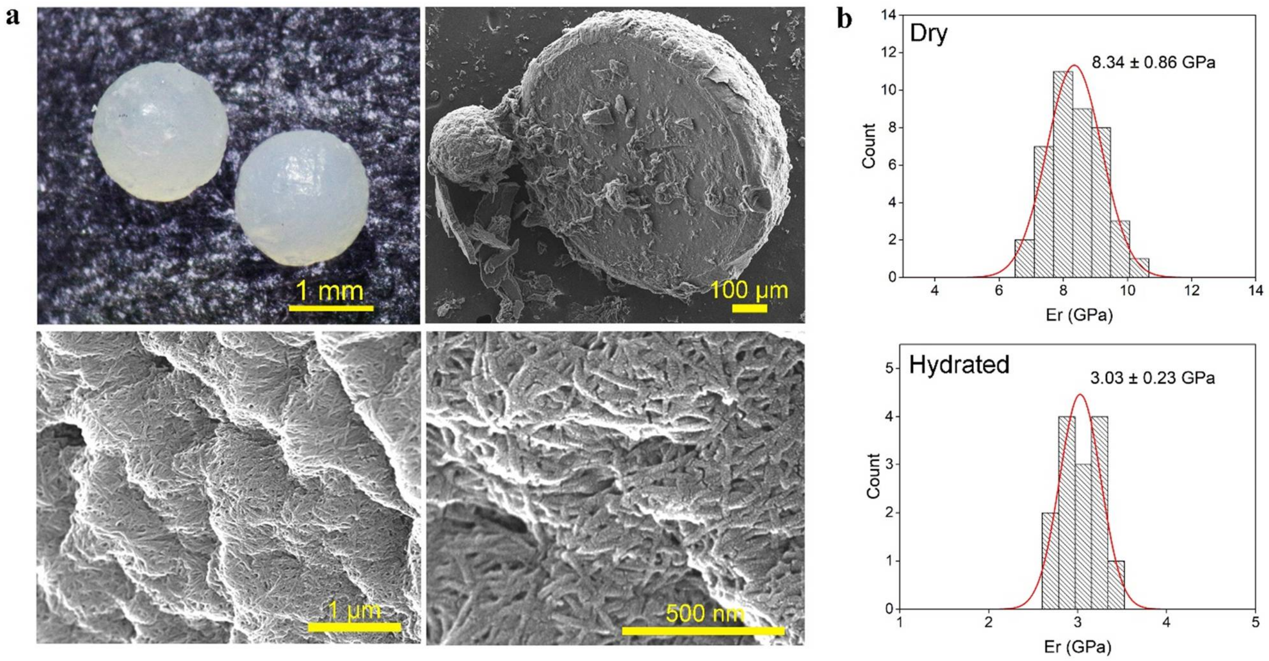

Figure S2. Bead-like structures formed by **GL8** peptide via self-assembly in DI water. a) Optical image of 2 beads formed by **GL8** peptide and SEM images showing the morphology and fibrous macro-scale assembly of the beads. b) Young's modulus obtained of **GL8** beads performed via nanoindentation in dried and hydrated conditions were 8.34 GPa and 3.03 GPa respectively.

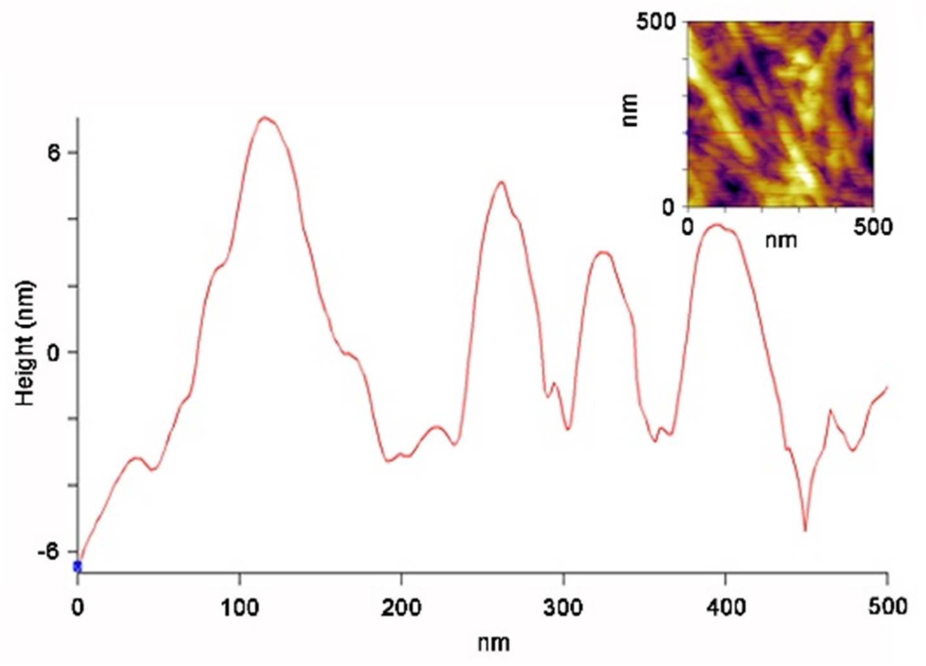

Figure S3. Topology of **GV8** hydrogel**.** Surface roughness plot across height profiles of dried **GV8** hydrogel samples measured via AFM revealed fibers of *ca.* 5-10 nm height.

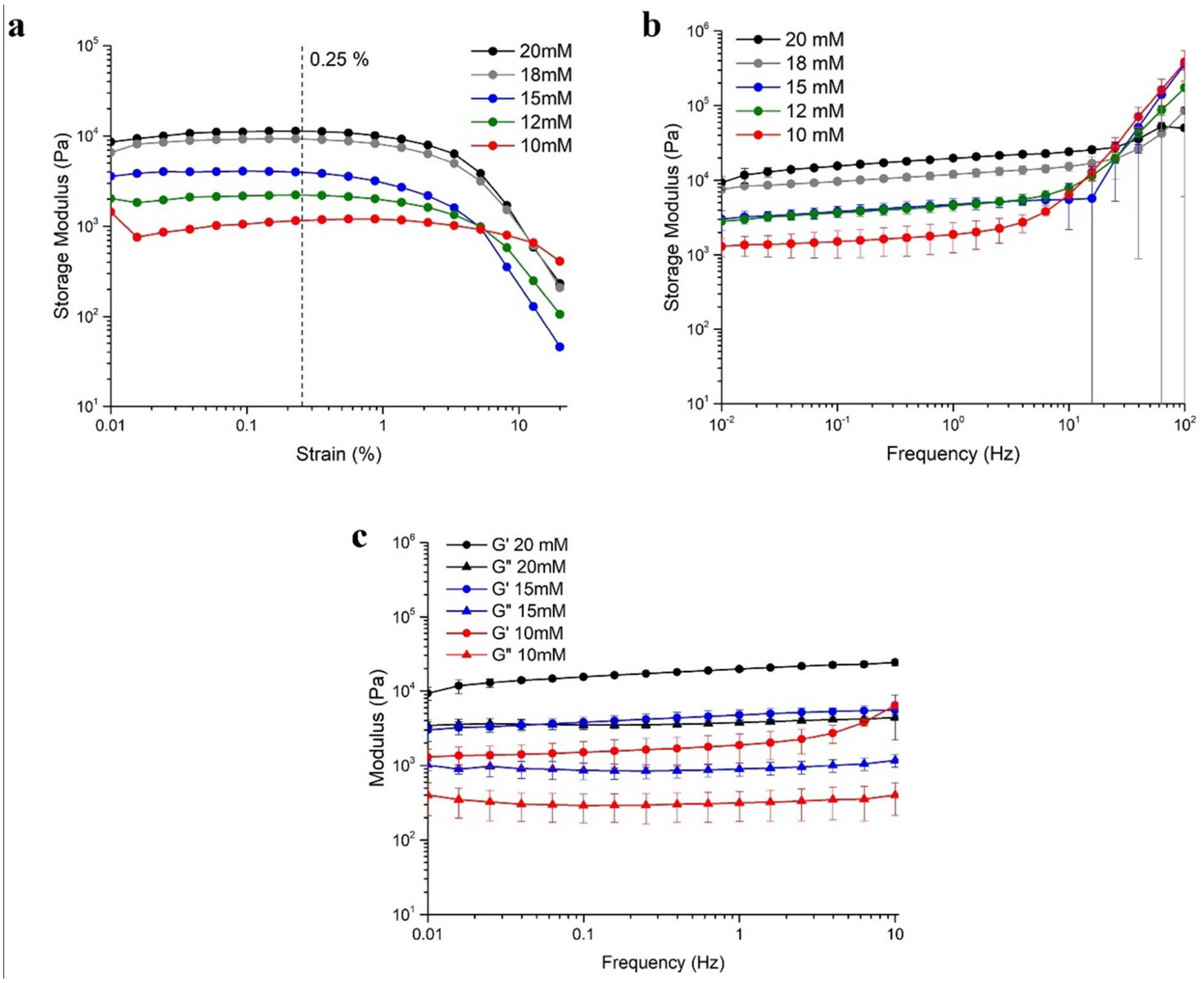

Figure S4. Rheological measurements of **GV8** hydrogels. a) Amplitude sweeps were performed on **GV8** hydrogels of different concentrations (20 mM, 18 mM, 15 mM, 12 mM and 10 mM) to identify their linear viscoelastic region (LVE). b) storage modulus, G', obtained via frequency sweeps performed at 0.25 % strain with $n = 3$. c) plots of 3 **GV8** hydrogels at concentrations 20mM, 15mM and 10mM illustrating their gel characteristics G' > G" (loss modulus).

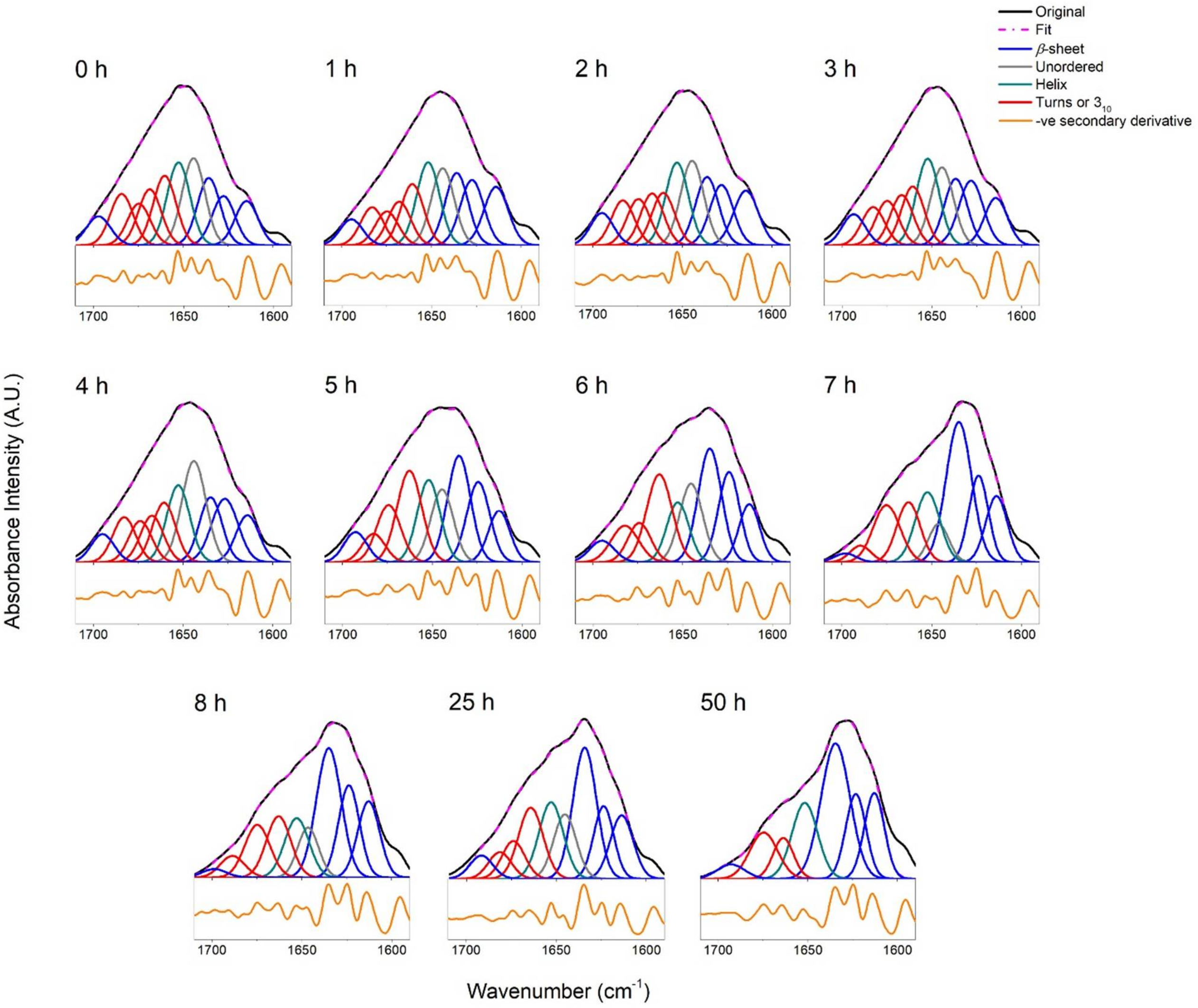

Figure S5. Deconvolution of ATR-FTIR spectra of **GV8** self-assembly over a time period of 50 h. Secondary derivatives were obtained to deconvolute the amide I bands of each spectra. FWHM of each fitted peak were kept consistent and positions of peaks were assigned accordingly.

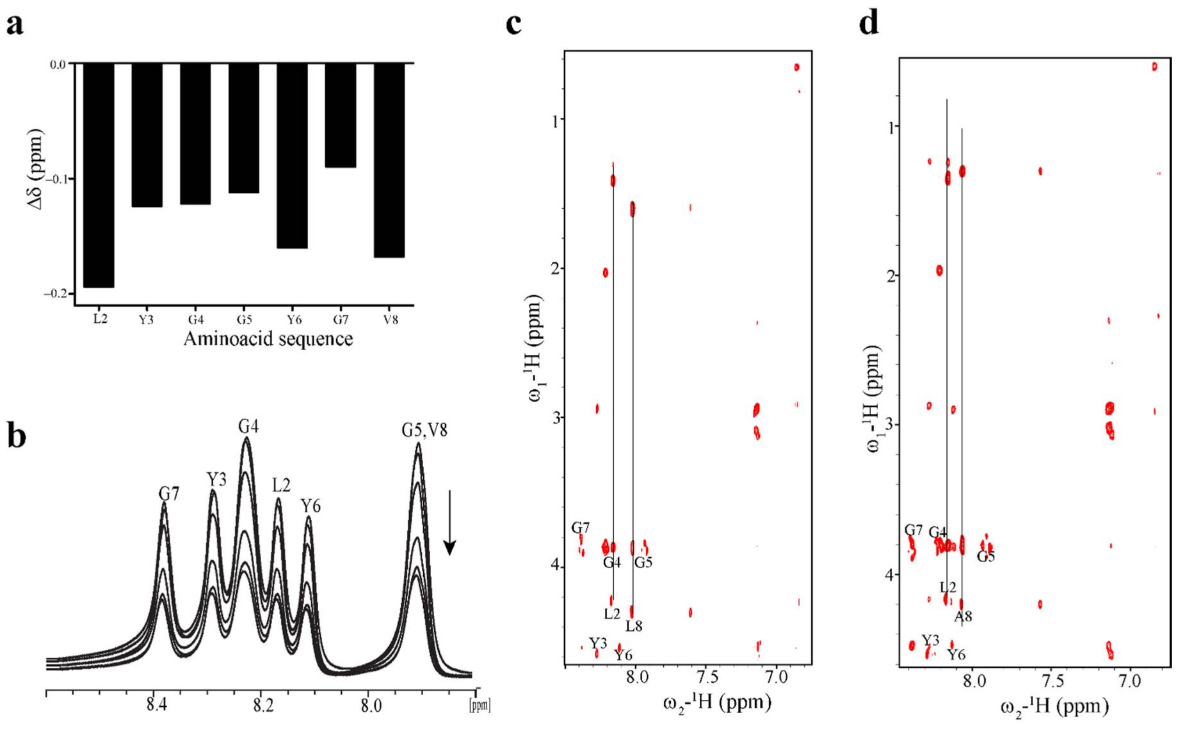

Figure S6. Chemical shift deviations (CSD) and gelation of **GV8** and 2D ^{1}H-^{1}H NOESY spectra of terminal mutated analogs of **GV8**. a) CSD plot of H^{α} values from random coil of **GV8** hydrogel. b) One dimensional ^{1}H spectra of 20 mM **GV8** peptide as function of time (every 4 h) for 18 hours. c,d) 2D ^{1}H-^{1}H NOESY spectra at 0.5 mM peptide concentration of **GL8** (c) and **GA8** (d) peptides indicating the absence of aromatic side chain interactions at 7.5-7.0 ppm.

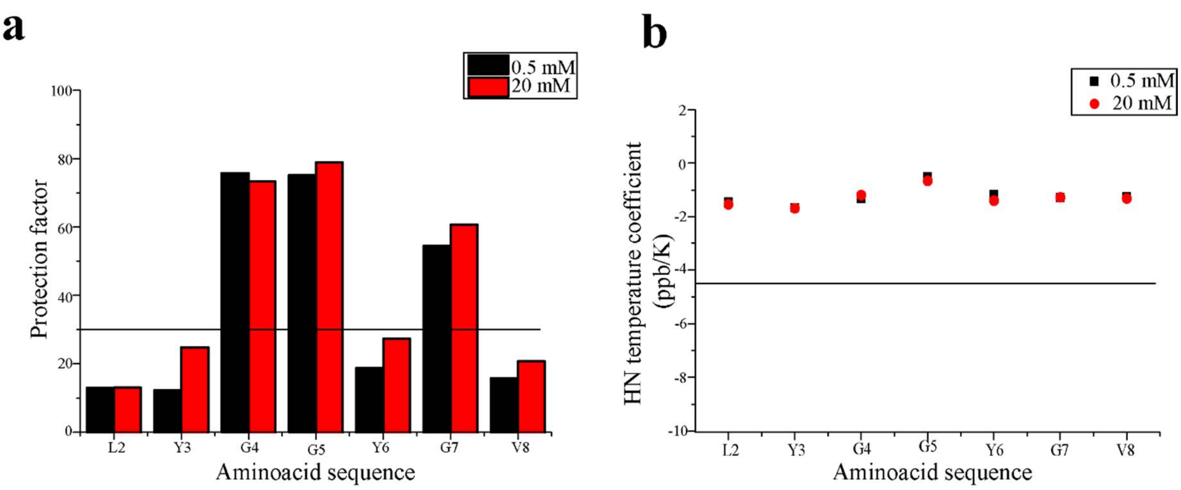

Figure S7. Validation of hydrogen bonds stabilization by H/D exchange NMR and Temperature coefficient parameter. a) H/D exchange protection factors for individual residues of **GV8** peptide at 0.5 and 20mM concentrations. b**)** Amide proton temperature coefficients of individual amino acids of **GV8** peptide at 0.5 and 20 mM concentrations.

Table S1. Heat-map summary of secondary structure assignments and their percentage composition from the deconvolution of Amide I peaks of **GV8** peptide over 50 h of incubation.

Assignment (%)	20mM peptide incubation duration										
	0 h	1 h	2 h	3 h	4 h	5 h	6 h	7 h	8 h	25 h	50 h
β-sheet	33.1	40.3	37.9	37.5	36.0	43.8	46.4	54.4	54.6	49.9	65.5
Unordered	15.0	13.7	14.6	13.7	18.3	11.8	13.4	6.1	8.1	11.2	-
α-Helix	14.3	14.7	14.1	15.2	13.1	13.4	9.7	13.5	11.2	14.3	16.4
Turns or 3_{10}	37.6	31.3	33.4	33.7	32.6	30.9	30.4	25.9	26.1	24.7	18.0

Supplementary Movie 1

GV8 peptide after overnight gelation, illustrating the gel structure and mechanical robustness.
